# Molecular Factors Predicting Ovarian Chemotoxicity in Fertile Women: A Systematic Review

**DOI:** 10.3390/cancers16162793

**Published:** 2024-08-08

**Authors:** Diego Raimondo, Antonio Raffone, Daniele Neola, Federica Genovese, Antonio Travaglino, Alberto Aguzzi, Valeria De Gobbi, Agnese Virgilio, Sara Di Santo, Rossella Vicenti, Valentina Magnani, Maurizio Guida, Tommaso Pippucci, Renato Seracchioli

**Affiliations:** 1 Division of Gynecology and Human Reproduction Physiopathology, IRCCS Azienda Ospedaliero-Universitaria di Bologna, 40138 Bologna, Italyalberto.aguzzi@studio.unibo.it (A.A.); valeria.degobbi@studio.unibo.it (V.D.G.); sara.disanto@studio.unibo.it (S.D.S.); rossella.vicenti@unibo.it (R.V.); valentina.magnani@unibo.it (V.M.); renato.seracchioli@unibo.it (R.S.); 2 Department of Woman, Child, and General and Specialized Surgery, University of Campania “Luigi Vanvitelli”, 80138 Naples, Italy; 3 Department of Medical and Surgical Sciences (DIMEC), University of Bologna, 40138 Bologna, Italy; tommaso.pippucci@unibo.it; 4 Department of Neuroscience, Reproductive Sciences and Dentistry, School of Medicine, University of Naples Federico II, 80131 Naples, Italy; federica.genovese@unina.it (F.G.); mauguida@unina.it (M.G.); 5 Unit of Pathology, Department of Medicine and Technological Innovation, University of Insubria, 21100 Varese, Italy; antonio.travaglino@uninsubria.it

**Keywords:** chemotherapy-induced ovarian failure, genetic markers, fertility preservation, ovarian damage, personalized medicine

## Abstract

**Simple Summary:**

This article explores the impact of chemotherapy on ovarian function in female cancer survivors, emphasizing the importance of preserving fertility alongside cancer treatment. Chemotherapy-induced ovarian failure (CIOF) poses a significant concern, affecting patients’ quality of life. The study aims to identify genetic markers predictive of CIOF through a systematic review of existing literature. The review identifies four relevant studies focusing on genetic factors associated with CIOF. Findings suggest potential associations between genetic variations, like CYP3A4*1B and GSTA1, and CIOF risk. Moreover, BRCA mutations may influence ovarian reserve recovery post-chemotherapy. While current assessment methods rely on biochemical tests and ultrasound imaging, genetic testing holds promise for personalized fertility preservation strategies. Integrating genetic markers into clinical practice could revolutionize decision making in fertility preservation for female cancer survivors, enhancing reproductive potential preservation.

**Abstract:**

**Background**: Recent advances in cancer diagnosis and treatment have significantly improved survival rates among women of reproductive age facing cancer. However, the potential iatrogenic loss of fertility caused by chemotherapeutic agents underscores the need to understand and predict chemotherapy-induced ovarian damage. This study addresses this gap by systematically reviewing the literature to investigate genetic markers associated with chemotherapy-induced ovarian failure (CIOF). **Objective**: The primary objective is to identify genetic markers linked to CIOF, contributing to a comprehensive understanding of the factors influencing fertility preservation in female cancer survivors. **Methods**: A systematic review was conducted using PubMed, EMBASE, Web of Science, Scopus, and OVID electronic databases from inception through December 2023. Studies were included if they featured genomic assessments of genes or polymorphisms related to CIOF in women with histologically confirmed tumors. Exclusion criteria comprised in vitro and animal studies, reviews, and pilot studies. The resulting four human-based studies were scrutinized for insights into genetic influences on CIOF. **Results**: Of the 5179 articles initially identified, four studies met the inclusion criteria, focusing on alkylating agents, particularly cyclophosphamide, and anthracyclines. Su et al. explored *CYP3A41B* variants, revealing modified associations with CIOF based on age. Charo et al. investigated *GSTA1* and *CYP2C19* polymorphisms, emphasizing the need to consider age and tamoxifen therapy in assessing associations. Oktay et al. delved into the impact of BRCA mutations on anti-Müllerian hormone (AMH) levels post-chemotherapy, supported by in vitro assays. Van der Perk et al. focused on childhood cancer survivors and revealed significant associations of *CYP3A43* and CYP2B6*2 SNPs with AMH levels. **Conclusions**: This systematic review analyzes evidence regarding genetic markers influencing CIOF, emphasizing the complex interplay of age, specific genetic variants, and chemotherapy regimens. The findings underscore the need for a personalized approach in assessing CIOF risk, integrating genetic markers with traditional ovarian reserve testing. The implications of this study extend to potential advancements in fertility preservation strategies, offering clinicians a comprehensive baseline assessment for tailored interventions based on each patient’s unique genetic profile. Further research is essential to validate these findings and establish a robust framework for integrating genetic markers into clinical practice.

## 1. Introduction

Recent advances in the diagnosis of cancer and the introduction of new protocols of chemo- and radiotherapy have significantly increased the survival rates of women of reproductive age dealing with cancer. However, the efficacy of anticancer treatments in extending life expectancy can be considered truly successful only when the preservation of patients’ quality of life is ensured. Recent studies indicate that the potential iatrogenic loss of fertility due to chemotherapy profoundly affects young women and may surpass the stress of the cancer diagnosis itself [1,2]. Preserving the ovarian function and the ability to conceive biological children appears as a paramount objective for numerous cancer survivors [3]. 

Indeed, chemotherapy and radiotherapy can severely affect or, in some cases, totally destroy the reproductive potential of patients, inducing premature ovarian insufficiency (POI) [4], since they damage primordial ovarian follicles and thus reduce follicular pool [5,6,7,8].

Generally, chemotherapy-induced ovarian failure (CIOF) was defined as the presence of either amenorrhea for 12 months, or follicle stimulating hormone (FSH) levels in the post-menopausal range, often at 6 months to 2 years after chemotherapy, or a combination of these [9].

**Figure 1:**
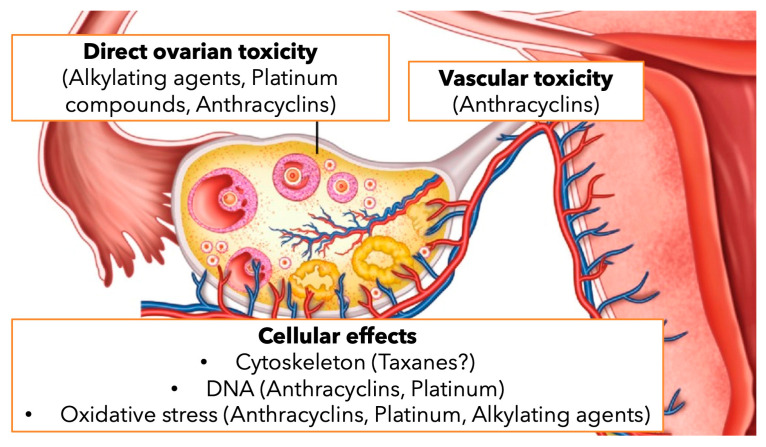
Gonadotoxic chemotherapy agents and suggested mechanisms for ovarian toxicity.

Several mechanisms by which chemotherapy drugs may damage the ovary were described: prenatal loss of oogonia, direct loss of primordial follicles, accelerated activation of primordial follicles, follicular atresia, stromal tissue damage, and damage to the vasculature or inflammation [10,11] (Figure 1). The most gonadotoxic chemotherapeutic agents are alkylating agents (such as cyclophosphamide) and anthracyclines (such as doxorubicin) [12].

However, the ovarian reserve reduction post-chemotherapy might be influenced by various factors, such as age, cancer type and severity, baseline ovarian reserve before treatment, and the specific treatment regimen. As women age and ovarian reserve naturally diminishes, the remaining follicular pool becomes more susceptible to depletion and damage, heightening the risk of premature ovarian failure following chemotherapy [13]. 

Objective parameters to define CIOF [14] are follicle stimulating hormone (FSH) and serum estradiol; on the other hand, the ovarian reserve can be assessed through anti-Mullerian hormone (AMH) and antral follicle count (AFC) at ultrasound imaging [15]. Unfortunately, these methods have the disadvantage of not accurately predicting future reproductive potential for patients undergoing chemotherapy. 

Nonetheless, numerous researchers have delved into the molecular mechanisms underlying chemotherapy-induced ovarian damage. Comprehending the molecular causes of treatment-induced ovarian dysfunction holds promise in pinpointing targets for mitigating and preventing gonadal damage during cancer therapy, thereby expanding the array of fertility preservation options available [16]. 

The aim of our study is to assess the existing literature investigating genetic markers able to predict chemotherapy-induced ovarian damage, through a systematic review of the literature. 

The identification of genetic markers associated with CIOF could be used, in addition to ovarian reserve testing, to provide clinicians with a comprehensive gynecological baseline condition assessment of fertility, in order to build a patient-tailored approach before chemotherapy. 

## 2. Materials and Methods

### 2.1. Study Protocol

Each step of this systematic review was defined a priori and described in a protocol registered in the PROSPERO international database of prospectively registered systematic reviews (identification code: CRD42024507064). Every stage of this review (i.e., search strategy, study selection, risk of bias assessment, data extraction, and data analysis) was performed by two authors independently. Cases of disagreement were resolved by discussion among all authors. 

We adopted the Preferred Reporting Item for Systematic Reviews and Meta-analyses (PRISMA) statement and checklist for reporting the study [17]. 

### 2.2. Search Strategy

A literature search was conducted by two authors independently in PubMed, EMBASE, Web of Science, Scopus and OVID electronic databases using a combination of the following text words and MeSH terms: “gonad*”, “ovar*”, “toxicity”, “impairment”, “failure”, “dysfunction”, “chemotherapy”, “chemotherapy-induced”, “cancer treatment”, “cancer survivor”, “genetic”, “polymorphism”, “mutation”, “genetic variation”, “SNP”, “single nucleotide polymorphism”, “GWAS”, “genome wide association studies”, “genome” from the inception of each database through December 2023. 

References list from each eligible study were also screened for missed studies.

### 2.3. Study Selection

We included all studies that reported evidence regarding women with histologically confirmed solid or liquid tumors for which chemotherapy was indicated, who had a genomic assessment for genes or polymorphisms of genes correlated to CIOF. We excluded in vitro and animal-based studies, reviews, pilot studies, case series, case reports, and video articles. We also excluded a priori studies in languages other than English.

### 2.4. Data Extraction

Data extraction was performed without modification of the original data. For each included study, we collected the following data:

- Type of study (observational or interventional design, retrospective or prospective data, randomized or non-randomized allocation of patients);
- Country
- Number of patients
- Genes associated with CIOF assessed in the study
- Chemotherapy drugs studied
- Type of neoplasm

### 2.5. Assessment of Risk of Bias within Studies

To assess the risk of bias within studies, we used the Methodological Index for Non-Randomized Studies (MINORS) [18]. For each study, we assessed 7 domains related to risk of bias: (1) Aim (if the study had a clearly stated aim); (2) Patient selection (if patients were consecutively or randomly selected for inclusion in the study); (3) Prospective data collection (if data collection followed an a priori defined study protocol); (4) Appropriate endpoints (if the assessment of CIOF was present); (5) Unbiased assessment of the study endpoint (if the methods of assessment of CIOF were clearly described, objective and reproducible); (6) Appropriate follow-up period (if the follow-up was at least 24 months, as for the definition of CIOF [9]); (7) Loss to follow-up (if patients lost to follow-up were less than 5% of the total study population).

Authors judged each of the included studies at “low risk of bias” if data about the domain were “reported and adequate”, “unclear risk of bias” if data about the domain were “not reported”, or “high risk of bias” if data about the domain were “reported but inadequate”.

## 3. Results

### 3.1. Study Selection

Our research through electronic databases searches identified 5179 articles, of which 1345 articles remained after duplicate removal and 289 after title screening. After abstract screening, we reviewed all the studies (n = 35) which reported evidence of genetic factors affecting chemotherapy-induced ovarian toxicity.

**Figure 2:**
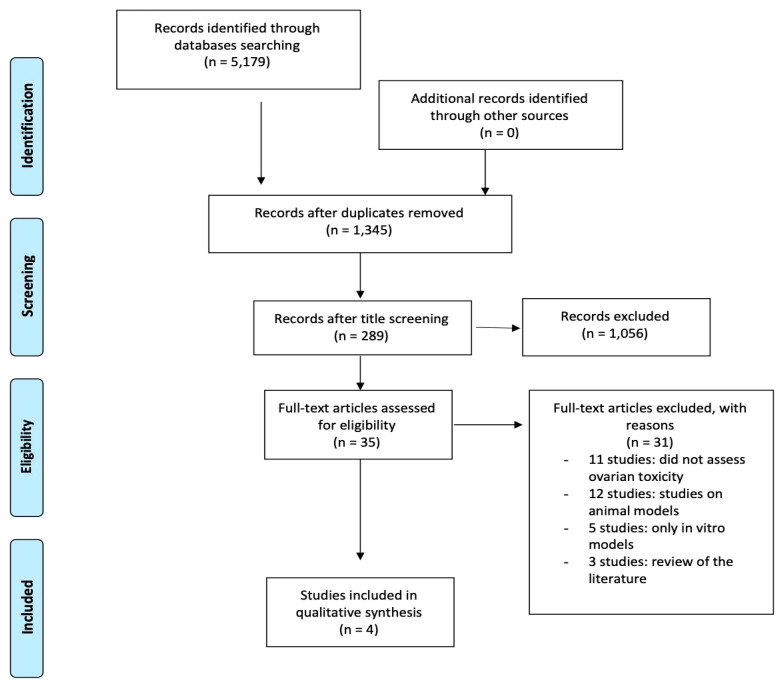
Flowchart of study selection step of the systematic review and meta-analysis (Prisma template [17]).

Excluding all the in vitro and animal-based studies, reviews, studies which did not assess ovarian toxicity, and selecting only studies on human samples, we finally obtained 4 articles concerning the impact of genetic factors on chemotherapy-induced ovarian toxicity [19,20,21,22] (Figure 2).

### 3.2. Studies and Patients’ Characteristics

The principal features of these studies are resumed in Table 1. The articles were published between 2010 and 2021: three studies [19,20,21] had a population sample from USA, while only one study [22] was the result of a 13 countries cooperating in an international project, part of the Pan Care LIFE initiative [23]. The studies by Su et al., Charo et al. and Oktay et al. were designed as prospective cohort studies; otherwise, the study of van der Perk et al. analyzed two different populations: the “discovery” cohort from Pan Care LIFE (a retrospective study) and the “replication” cohort from SJLIFE (a retrospectively-constructed cohort with prospective follow-up) [24], with the latter being used to validate the results and to perform a meta-analysis of the former. 

The entire population from the studies of Su et al., Charo et al. and Oktay et al. was affected by histologically confirmed breast cancer, while the study of van der Perk et al. included a large variety of cancers, including leukemia, lymphoma, brain tumor, neuroblastoma, renal tumor, carcinoma, osteosarcoma, Ewing sarcoma, germ cells tumor, skin cancer, retinoblastoma and others. All 4 studies included patients that underwent alkylating agent-based chemotherapy schemes (specifically cyclophosphamide-based); the specific schemes used in each study are summarized in Table 1.

The main outcome used to establish an association between genetic variation and CIOF varied among those studies: Su et al. and Charo et al. used “time to CIOF” as primary outcome and used patients’ self-reported menstrual bleeding diaries to determine this, defining CIOF as 12 months of amenorrhea after the beginning of chemotherapy; Charo et al. also added clinical questionnaires every three to six months; on the other hand, Oktay et al. and van der Perk et al. analyzed anti-Müllerian hormone (AMH) serum level as a biochemical marker for ovarian function and reserve after chemotherapy treatment.

All the genes and Single Nucleotide Polymorphisms (SNPs) analyzed by the included studies were selected based on the evidence of their association with cancer outcomes or with ovarian function after chemotherapy exposure (Table 1). Su et al. studied SNPs in genes involved in both cyclophosphamide activation and detoxification. The median follow-up period was 5.2 years and 51% of the entire population developed CIOF. Median age at chemotherapy was 43.2 years. No significant association was found between CIOF and selected SNPs. However, the CIOF and SNP association was modified by age at chemotherapy. Patients with CYP3A4*1B variants of age < 45 at the time of chemotherapy had significantly longer time to CIOF than patients homozygote for CYP3A4*1A (HR 0.25 [95% CI 0.07–0.9]). In addition, age and tamoxifen use were also found to be independently associated with CIOF. The entire population of this study was dichotomized using an age, setting threshold at 45, because ovarian failure occurring at less than 45 years old is considered as early menopause and pathologic [19].

Charo et al. considered SNPs in genes involved in the metabolism of cyclophosphamide, in particular in its activation and detoxification. The median follow-up period was 808 days and 28% of the population experienced CIOF. Median age at chemotherapy was 39.7 years. Patients who resulted as homozygous for the GSTA1 minor allele were less likely to develop CIOF less (HR 0.22 [95% CI 0.05–0.94], *p* = 0.04), while women homozygous for the CYP2C19 minor allele could develop CIOF with a higher probability (HR 4.5 [95% CI 1.5–13.4], *p* = 0.007) than women with at least one major allele. However, the adjustment of the analysis for age and tamoxifen use showed that the associations were no longer statistically significant (GSTA1: HR 0.24 [95% CI 0.06–1.0], *p* = 0.05; CYP2C19: HR 2.5 [0.8–7.6], *p* = 0.11) [20].

Oktay et al. studied BRCA mutation as a possible risk factor for CIOF. Serum AMH levels were collected at baseline (before chemotherapy) and 12, 18 and 24 months after the treatment. Median age at chemotherapy was 35.8 years. It was found that patients who had a BRCA mutation had a three-times difference in AMH recovery after chemotherapy (1.6%), when compared with BRCA negative (3.7%) and untested (= low familiar risk) controls (5.2%). In a sub-analysis limited to doxorubicin + cyclophosphamide + paclitaxel therapy, the most common chemotherapy regimen, the results were similar. Furthermore, these results were confirmed within the same study through an in vitro BRCA—knockdown mouse oocyte bioassay, demonstrating that BRCA deficiency was linked to heightened susceptibility of oocytes to doxorubicin [21].

Finally, van der Perk et al. studied only SNPs in genes involved in cyclophosphamide activation and this was the only included study to collect a population of women who underwent chemotherapy during pediatric age, defining these young women as Childhood Cancer Survivors (CCSs). In this study, CYP3A4*3 SNP was found to be significantly associated with decreased AMH levels. The authors performed further analyses, discovering a significant main deleterious effect (*p* = 7 × 10^−4^) of CYP3A4*3 (rs4986910) on log-transformed AMH levels, while CYP2B6*2 SNP (rs8192709) had a significant protective interaction effect (*p* = 0.01) on log-transformed AMH levels in CCSs, receiving more than a 8000 mg/m^2^ Cyclophosphamide Equivalent Dose (CED) [22]. 

### 3.3. Risk of Bias Assessment

For the “Patient selection” domain, three studies [19,20,21] were judged as showing unclear risk of bias because they did not report if patients were randomly or consecutively included. For the “Loss to follow-up” domain, two studies [19,21] were judged at high risk of bias, since the loss of patients at follow-up was >5%, while two studies [20,22] were considered at unclear risk of bias because they did not report the number of patients lost at follow-up. All included studies were judged at low risk of bias for the other domains (Table 2).

## 4. Discussion

Our systematic review investigated genetic markers that could serve as predictors for chemotherapy-induced ovarian damage. The identification of four pertinent articles provided a foundation for understanding the genetic underpinnings of CIOF across different chemotherapy regimens.

The selected studies primarily focused on alkylating agents, particularly cyclophosphamide, and anthracyclines, such as doxorubicin, known for their pronounced gonadotoxic effects. This aligns with existing literature highlighting the detrimental impact of these agents on ovarian function [12].

Since three out of four included studies regarded women with breast cancer, and chemotherapy regimens were similar in all included studies, a robust analysis of genetic influences on ovarian function post-chemotherapy was feasible. The choice of outcome measures, ranging from time to CIOF to anti-Müllerian hormone (AMH) levels, added a layer of depth to our understanding of genetic influences on ovarian function post-chemotherapy.

Su et al.’s investigation of SNPs in genes related to both cyclophosphamide activation and detoxification yielded intriguing findings. The nuanced interplay between age, specific genetic variants (CYP3A4*1B), and tamoxifen use demonstrated the importance of personalized considerations in assessing CIOF risk. Similarly, Charo et al.’s exploration of *GSTA1* and *CYP2C19* polymorphisms underscored the need to account for confounding factors, like age and tamoxifen therapy, revealing complex associations.

The study by Oktay et al. delved into the role of *BRCA* mutations in CIOF, emphasizing the potential three-fold difference in AMH recovery after chemotherapy among patients with BRCA mutations. The incorporation of in vitro assays lent further credence to these findings, offering a mechanistic understanding of *BRCA* deficiency’s impact on oocyte susceptibility to doxorubicin.

Notably, van der Perk et al.’s unique focus on SNPs in genes involved in cyclophosphamide activation within a population of Childhood Cancer Survivors (CCSs) highlighted the importance of considering age at chemotherapy. The identification of *CYP3A43* and *CYP2B62* SNPs associated with AMH levels in CCSs presented valuable insights into the long-term effects of chemotherapy administered during pediatric age.

From our review, it seems that genetic polymorphisms in cytochrome P 450 (CYP450) and Glutathione S-transferase A 1 (GSTA1) enzymes are most likely correlated with the risk of CIOF. This could be explained by the fact that alkylating agents are metabolized by CYP450 and GSTA1 enzymes, therefore alterations of their functioning may influence the pharmacokinetics of these drugs and their effects on ovarian tissue, in negative as in *CYP2C19* minor allele, or in positive, as in *GSTA1* minor allele [20]. The study by Oktay et al. was the only one assessing the role of *BRCA* mutations in the risk of CIOF. Such an association may be explained by the assumption that alkylating agents induce DNA double strand breaks (DSBs) in human primordial follicle oocytes: women with *BRCA* mutations have a deficit in DSB repair and thereby may have a higher susceptibility to chemotherapy-induced loss of ovarian reserve [21]. Another mechanism could be the connection between the *BRCA* mutation and diminished ovarian reserve and accelerated ovarian aging, beyond chemotherapy, which was described in several studies [25,26], but is still debated in the literature [27].

However, our research also highlights that other inter-individual factors, such as age at chemotherapy and tamoxifen therapy, could influence the risk of CIOF.

Healthcare providers attending to oncology patients should discuss the potential for infertility as soon as feasible before initiating treatment, as stated by the American Society of Clinical Oncology (ASCO) [28], since chemotherapy agents can damage the ovarian tissue and induce POI. 

In recent times, there has been a rising incidence of iatrogenic POI among women with cancer: approximately 4% of fertile patients are diagnosed with malignancy and face potentially sterilizing gonadotoxic treatments, such as chemotherapy [29,30]. Chemotherapy induces apoptosis of the primordial follicle pool, damage to the ovarian cortex, and reduces ovarian blood flow [31]. 

In light of the rising incidence of iatrogenic premature ovarian insufficiency (POI) among cancer patients, fertility preservation strategies assume paramount importance. Patients with the desire for offspring can choose between two different main methods of fertility preservation before gonadotoxic cancer treatments: oocyte and/or ovarian tissue cryopreservation (OTC) [32,33]. Oocyte cryopreservation is the extraction and maturation of immature oocytes in vitro; the matured oocytes can subsequently undergo insemination, and the resulting embryos can be cryopreserved or vitrified for future utilization [34,35]. However, it has three important limitations: first, ovarian stimulation for the retrieval of mature oocyte cryopreservation can delay the start of cancer treatment; second, in some hormone dependent cancers, ovarian stimulation should be avoided; third, it cannot be performed in pre-pubertal patients [36]. In such scenarios, OTC serves as a viable strategy to safeguard endocrine and reproductive function in patients facing a high risk of CIOF [36,37]. This method involves laparoscopic surgery to extract either parts of the ovarian cortex or the entire ovary for cryopreservation. Upon disease remission and when fertility is desired, the cryopreserved ovarian tissue can be re-implanted in the patient, either orthotopically into the ovarian fossa or within the atrophic ovarian cortex. The effectiveness of OTC and subsequent ovarian tissue transplantation is evidenced by high rates of ovarian function recovery (95% of cases), numerous live births (exceeding 200 worldwide to date), and successful induction of puberty [36,37]; however, it is very expensive in the present model [38]. Therefore, the most appropriate way to maximize cost-effectiveness would be to increase the number of patients who preserve ovarian tissue that actually undergo ovarian tissue transplant after 5 years. To reach this goal, it appears crucial to perform a detailed patient selection with an accurate analysis of gynecological baseline condition before starting the protocol, carefully counselling patients in order to make them understand their baseline and predicted post-chemotherapy reproductive potential [39].

This requires the application of clinical markers that can accurately predict individual susceptibility to CIOF. Current ovarian reserve assessment mainly relies on biochemical tests and ultrasound imaging. Objective parameters to define chemotherapy-induced ovarian failure [14] are follicle-stimulating hormone (FSH) and serum estradiol serum; while anti-Mullerian hormone (AMH) and antral follicle count (AFC) at ultrasound imaging are used to define the ovarian reserve [15]. Unfortunately, these methods do not accurately predict future reproductive potential for patients undergoing chemotherapy. In our research, different studies assessed CIOF by analyzing different parameters (in particular time to CIOF, AMH serum levels, clinical questionnaires), highlighting the impossibility of adequately comparing studies, and the need to establish a standardized evaluation of ovarian reserve after chemotherapy. However, to the best of our knowledge, it appears unclear if any parameter is more reliable than the others when defining CIOF.

Numerous researchers have examined the molecular mechanisms responsible for CIOF and how to prevent it, with both animal models and in vitro studies. Zhao et al. [40] found that hyaluronic acid has the ability to prevent immunosuppressive drug-induced ovarian damage in rats. Huang et al. [41] investigated the effects of metformin on cyclophosphamide-induced POI and observed that concurrent metformin treatment during cyclophosphamide therapy could significantly preserve ovarian function and fertility in mice. Delkhosh et al. [42] also studied cyclophosphamide-induced POI in a mouse model and the effects of coenzyme Q10 administration, highlighting that this coenzyme has a positive impact on the reproductive system due to its antioxidant properties, protecting the cells from free-radical oxidative damage and apoptosis. Lastly, a protective effect was demonstrated of administration of SB216763, a small and potent glycogen synthase kinase-3 inhibitor, against doxorubicin-induced oxidative damage to mouse ovarian reserve [43]. Such results in animal and in vitro models need to be confirmed in humans before being introduced to clinical practice. However, they represent important steps towards the understanding of the molecular mechanisms of CIOF.

Grasping CIOF molecular origins can help to identify targets to prevent and mitigate gonadal damage during cancer treatment, thereby expanding fertility preservation options [16]. In this sense, the adoption of genetic testing, including next-generation sequencing, seems to have great potential in understanding the genomic aspects of chemotherapy-induced ovarian insufficiency. The exploration of genes implicated in CIOF might also be extended to examining somatic characteristics of ovarian tissue post-cryopreservation. This avenue of investigation suggests that next-generation sequencing holds significant promise in elucidating the genomics of CIOF. By embracing the concept of precision medicine, which tailors treatment strategies to individual patients, we may enhance our ability to combat cancer while safeguarding fertility. By employing genetic testing techniques, both at the germinal and somatic levels (such as on cryopreserved ovarian tissue), clinicians could potentially identify patients with gonads susceptible to chemotherapeutic agents. This personalized approach enables the development of tailored fertility preservation strategies for each patient. As research continues to unravel the genomic alterations associated with CIOF, mutations affecting relevant molecular pathways may offer valuable insights into reproductive potential [16].

Despite the high predictive value of such genetic tests in high-risk populations, they are not yet routinely utilized in clinical settings for the general population. However, there is growing interest in leveraging genetic markers to forecast ovarian function outcomes following chemotherapy. An editorial has even proposed the integration of genetic testing into clinical practice to better inform fertility preservation decisions [44]. This highlights the evolving landscape of precision medicine in oncology and underscores the potential of genetic testing to revolutionize fertility preservation strategies in cancer patients.

This study has the strength of providing a thorough investigation into the molecular mechanisms of CIOF in humans, contributing insights to the field. By identifying potential targets for preventing gonadal damage, this research has direct implications for improving fertility preservation in cancer patients and stimulating research about this topic. Our research strategy has the limitation of not including any in vitro and animal studies, but animal models and in vitro studies cannot fully replicate the complexity of human ovarian physiology and genetic interactions, do not encompass the genetic diversity present in human population, do not take into account the complex interactions between various genetic, environmental, and hormonal factors that are unique to humans, and do not assess clinically relevant outcomes, such as fertility preservation and ovarian reserve, measured through specific biomarkers (e.g., AMH levels) and clinical endpoints (e.g., amenorrhea). Further studies appear paramount to validate our findings with independent analyses and provide tailored strategies to prevent CIOF in women affect by cancer.

## 5. Conclusions

In conclusion, our systematic review summarizes evidence from diverse studies, providing a foundation for understanding the genetic determinants of chemotherapy-induced ovarian damage. The findings underscore the complexity of these interactions and advocate for a personalized, multifaceted approach in assessing the risk of CIOF. The integration of genetic markers alongside traditional ovarian reserve testing could potentially revolutionize fertility preservation strategies, allowing clinicians to tailor interventions based on a comprehensive understanding of each patient’s unique genetic profile. As we move forward, further research and validation are warranted to establish a robust framework for implementing genetic markers in clinical practice, ultimately enhancing the preservation of reproductive potential in female cancer survivors.

## Figures and Tables

**Table 1:** Characteristics of the included studies.

Study	Country	Patients n	Studied Genes	Chemotherapy	**Type of Cancer**
2010 Su et al. [19]	USA	127	*CYP2B6*, *CYP2C9*, *CYP3A4*, *CYP3A5*, *GSTM1*, *GSTP1*, *GSTA1* and *GSTT1* (9 SNPs on a total of 8 DMEs)	Cyclophosphamide + Adriamycin ± (taxane OR 5-fluorouracil); others	Breast
2021 Charo et al. [20]	USA	115	*CYP3A4*, *CYP2C19*, *GSTP1* and *GSTA1*	Adriamycin+ cyclophosphamide ± paclitaxel ORDocetaxel + cyclophosphamide	Breast
2020 Oktay et al. [21]	USA	108	*BRCA1* and *BRCA 2*	Cyclophosphamide + doxorubicin + paclitaxel OR cyclophosphamide + methotrexate + 5-fluorouracile OR cyclophosphamide + epirubicina + docetaxel OR only Taxol; others	Breast
2021 van der Perk et al. [22]	International (PanCareLIFE project + SJLIFE study)	743 + 391	*CYP2C19*, *CYP3A4*, *CYP2B6* (9 SNPs on a total of 3 DMEs)	Alkylating agents [quantified using the CED-score]	Leukemia, HL, NHL, Brain, Neuroblastoma, Renal, Sarcomas, Germ Cell Tumor, Skin (incl. melanoma), Retinoblastoma, Liver, Thyroid, Colon, Other

DME: Drug Metabolism Enzyme; CED: Cyclophosphamide Equivalent Dose; HL: Hodgkin Lymphoma; NHL: Non-Hodgkin Lymphoma.

**Table 2:** Assessment of risk of bias within the included studies according to the Methodological Index for Non-Randomized Studies (MINORS). Summary of risk of bias for each study: Plus sign: low risk of bias; minus sign: high risk of bias; question mark: unclear risk of bias.

	Aim	Patient Selection	Prospective Data Collection	Appropriate Endpoints	Unbiased Assessment of the Study Endpoint	Appropriate Follow-Up Period	Loss to Follow-Up
2010 Su et al. [19]	+	?	+	+	+	+	−
2021 Charo et al. [20]	+	?	+	+	+	+	?
2020 Oktay et al. [21]	+	?	+	+	+	+	−
2021 Van Der Perk et al. [22]	+	+	+	+	+	+	?

## Data Availability

The data that support the findings of this study are available on request from the corresponding author.
